# CHEMICAL LEUKODERMA: WHAT’S NEW ON ETIOPATHOLOGICAL AND CLINICAL ASPECTS?

**DOI:** 10.4103/0019-5154.70680

**Published:** 2010

**Authors:** Sanjay Ghosh

**Affiliations:** *From the Departments of Dermatology, Venereology and Leprology, MGM Medical College and LSK Hospital, Kishanganj, Bihar, India*

**Keywords:** *Chemical leukoderma*, *contact leukoderma*, *occupational leukoderma*, *vitiligo*

## Abstract

Chemical leukoderma denotes an acquired hypopigmentation caused by repeated exposure to specific chemical compounds simulating clinically idiopathic vitiligo. The ailment has been increasing in developing countries like India in recent years. Etiologically, a lot of chemicals, especially phenolic group, have been identified in various countries including India. The term, “chemical leukoderma syndrome” has been described to encompass all the various manifestations of chemical leukoderma. Clinical diagnostic criteria have been proposed to diagnose chemical leukoderma clinically more confidently.

## Introduction

Chemical leukoderma represents an acquired vitiligo-like hypomelanosis induced by repeated exposure to specific chemical compounds. This chemical effect, independent of their sensitizing potential, is distinctly separate from post-inflammatory depigmentation and Koebner in vitiligo.[1]

Chemical leukoderma is also designated as contact leukoderma or occupational leukoderma. Chemical leukoderma has been reported from many countries of the world, developing as well as developed, induced probably by “globalization processes.”

Chemical leukoderma remains an under-diagnosed common condition often inducing clinical dilemma with idiopathic vitiligo in dermatological practice. This ailment has been increasing rapidly in incidence in recent decades in developing countries like India.[2]

## Etiological Aspects

The contributory chemicals are mostly aromatic or aliphatic derivatives of phenols and catechols.[3] Other responsible toxins are sulfhydryls, cinnamic aldehyde, *p*-phenylenediamine, mercurials, arsenics, azelaic acid, corticosteroids, tretinoin, otic preparations such as eserine and thiotepa as well as systemic medications such as chloroquine and fluphenazine (prolixin).[1,3,4] However, these chemicals are lethal for melanocytes only in persons having specific inherent susceptibility.[2,3]

Oliver *et al*. in 1939[5] first identified chemical leukoderma in a leather manufacturing company in workers who used “acid-cured” rubber gloves. Monobenzylether of hydroquinone (MBH), an antioxidant used in the rubber industry, was the culprit agent. In later decades, several reports of occupational leukoderma caused by phenolic compounds were published from various countries.[6] *Para*-tertiary butylphenol (PTBP) and *p*-tertiary octylphenol (PTOP) were reported offending chemicals from Japan,[7] whereas from Russia PTBP and *p*-tertiary butylphenolformaldehyde (PTBPF) resins were reported.[8] Occupational depigmentation in tappet assembly workers exposed to *p*-tertiary butylcatechol (PTBC) was mentioned by Gellin *et al*.[9] Depigmentation from certain phenolic detergent germicides such as PTBP and *p*-tertiary amylphenol (PTAP) was reported by Kahn.[10] Vitiligo caused by PTBP and homologues has been reported by Malten *et al*.[11] Chemical leukoderma from semipermanent and permanent hair colors and rinses was first published in 1993 by Taylor *et al*.[12] Offending chemicals were *para*-phenylenediamine (PPD) and benzyl alcohol. From India, chemical leukoderma was first reported by Pandhi and Kumar[13] induced by adhesive “bindi” (decorative color used on the forehead by Asian females) and “footwear.”[13] Bajaj *et al*. were the first to report chemical leukoderma from free PTBP in “bindi” adhesive,[14] from MBH in synthetic wallets causing depigmentation of the breast from the habit of keeping wallets inside blouses,[15] from MBH causing footwear depigmentation,[16] from PPD in hair dye,[17] from azo dye in “alta” (a decorative color used by Asian females on their feet)[18] and from solvent yellow 3, an azo dye, used in “alta,” and other domestic objects such as watch straps, spectacles, and hearing aids.[19]

Common offending agents responsible for chemical leukoderma are shown in Table 1. In a recent Indian publication etiological agents for chemical leukoderma identified were hair dye 27.4% (21% self-use; 6.4% not self-use), deodorant and spray perfume 21.6%, detergent and cleansers 15.4%, adhesive bindi 12%, rubber chappal 9.4%, black socks and shoes 9.1%, eyeliner 8.2%, lipliner 4.8%, rubber condoms 3.5%, lipstick 3.3%, fur toys 3.1%, toothpaste 1.9%, insecticides 1.7%, “alta” 1.2%, and amulet string color 0.9%.[2]

**Table 1:** Chemical leukoderma: contributory chemicals

Most potent phenol/catechol derivatives	
Monobenzyl ether of hydroquinone (MBH)	
Hydroquinone	
*p*-tert-Butylcatechol (PTBC)	
*p*-tert-Butylphenol (PTBP)	
*p*-tert-Amylphenol (PTAP)	
Additional phenol/catechol derivatives	
Monomethyl ether of hydroquinone (MMH)	
Monoethyl ether of hydroquinone (MEH)	
*p*-Phenylphenol	
*p*-Octylphenol	
*p*-Cresol	
Sulfhydryls	
Cysteamine	
Sulfanolic acid	
Cystamine dihydrochloride	
Miscellaneous	
Mercurials	Tretinoin
Arsenic	Benzoyl peroxide
Cinnamic aldehyde	Ammoniated mercury
PPD	Azelaic acid
Corticosteroids	Fluorouracil
Chloroquin	Briilliant lake red R
Soymilk and derived protein Thiotepa (inhibits PAR-2)	
(Miyamoto and Taylor[4] and Ortonne[1])	

In contrast to the western literature, in the above study, household chemical exposure was a much more common inducing agent than occupational chemical exposure. Nontechnical or nonindustrial occupations had more involvement than those in technical or industrial occupations. The probable reasons for these discrepancies of data between developed and developing countries could be: (1) lack of quality control in consumer products in developing countries (cheaper ingredients to compete in market even by multinational companies), (2) lack of reporting from industrial set up: (a) from owner’s view: fear of compensation, (b) from worker’s view: fear of loosing job, (c) from doctor’s view: lack of awareness, and (3) lack of industrialization.

## Etiopathogenesis

Predisposing factors for the development of chemical leukoderma predominantly constitutes genetic factors which render the melanocytes more fragile. Precipitating factors, which in comparison to idiopathic vitiligo are evident in chemical leukoderma, initiate programmed cell death, or apoptosis of melanocytes [Table 2].[3]

**Table 2:** Precipitating factors: Chemical leukoderma and vitiligo compared

Precipitating factors	
Known	Unknown
Specific cytotoxic factors	??
Chemical leukoderma	Vitiligo

Tyrosinase-related protein-1 (Tyrp1), by catalytic conversion of chemicals, produces radical oxygen species. This oxidative stress triggers activation of cellular free-radical scavenging pathway to prevent cell death. Genetic inability of melanocytes to tolerate and/or respond to oxidative stress may underlie etiology of chemical leukoderma.[4]

Elevated tumor necrosis factor related apoptosis-inducing ligands (TRAIL) death receptor expression and heat shock protein (HSP) play important role in onset of chemical leukoderma and later its generalization by systemic auto-immunity. Melanocyte exposed to 4-tertiary butyl phenol (TBP) induces elevated TRAIL expression. TRAIL expression is strongly positive even in peri-leukoderma skin.

**Figure 1:**
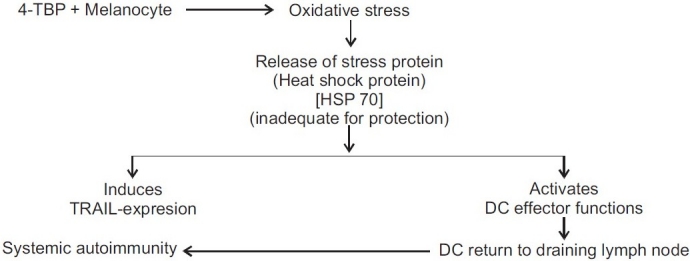
Pathogenesis of chemical leukoderma: elevated TRAIL and HSP^20^

Dendritic cells (DC) effector functions also take pivotal role in spreading of leukoderma. Stressed melanocytes mediate DC inactivation by releasing heat shock protein (HSP70). DC function is partially inhibited by antibody to TRAIL [Figure 1].[20]

## Clinical Features

Chemical leukoderma should be considered in the differential diagnosis of every case of idiopathic vitiligo or leukomelanoderma. Chemical leukoderma develops not only at the site of chemical contact, but also remotely. The mechanism responsible for this distant spread of the disease could be: (i) sensitization,[21] (ii) autotransfer, or heterotransfer of the chemical from patients themselves and people close to them.

Chemical leukoderma, such as vitiligo, lacks definitive diagnostic features. Clinico-histopathologically, no absolute criteria can differentiate chemical leukoderma from vitiligo. However, chemical leukoderma can be diagnosed clinically by a history of repeated exposure to a known or suspected depigmenting agent at the primary site, distribution of macules corresponding to chemical exposure, and the presence of numerous acquired confetti or pea-sized macules [Table 3].[21]

**Table 3:** Chemical leukoderma: Clinical comparison with vitiligo (Mosher[21] and Ortonne[1])

	Chemical leukoderma	Vitiligo
Trichome	–	+
Leukotrichia	–	+
Koebner	–	+
Confetti macules	+	–

Chemical leukoderma can be differentiated from post-inflammatory leukoderma by the clinical features of the latter: (1) primary rash always limited to the original area and (2) no confetti or remote macules.[21] Similarly, chemical leukoderma can be compared with “Koebner in vitiligo”by the following features in the latter: (1) history of single chemical exposure or trauma, (2) usually linear, and (3) pre-existing vitiligo.[21]

All age groups from pediatric to geriatric including neonates may be affected by chemical leukoderma. Although adults have a much higher incidence of chemical leukoderma, a considerable number of children below the age of 12 years may also be affected, which has not been reported much in the western literature. This may indicate that exposure to household objects rather than to industrial chemicals has caused an important role in the pathogenesis of chemical leukoderma in Indian patients. Females suffered more than males probably due to more exposure to offending household objects and more offering of treatment due to the prevalent social stigma regarding vitiligo and any other depigmenting skin diseases in Asian countries.[2]

The presence of numerous acquired confetti or pea-sized macules is characteristic, although not diagnostic, of chemical leukoderma. In an Indian study, a considerable number of patients (88.4%) showed confetti macules, which thus represents an important diagnostic clue for chemical leukoderma.[2]

Contact dermatitis is not a prerequisite for the development of chemical leukoderma.[21] However, in some cases of concurrent contact dermatitis and chemical leukoderma the same offending chemical may cause contact dermatitis as well as chemical leukoderma in some patients but by a different pathomechanism.

The face and scalp were the commonest and least affected sites involved, respectively, in chemical leukoderma. Within the face the eyelids were a major area of involvement. This probably originates from greater penetration of the offending toxic chemicals through the thinner skin of the face (eyelids being the thinnest) compared with the thicker skin of the scalp. However, the hands and feet, although composed of much thicker skin, showed a high incidence of chemical leukoderma, probably due to a higher rate of exposure.[2]

## Chemical Leukoderma or Contact Leukoderma or Occupational Leukoderma?

There prevails some confusion regarding the varying terminology of chemical leukoderma or occupational leukoderma. The term “contact leukoderma” may be confusing as the adjective “contact” may indicate that leukoderma is confined only to the site of contact, which is baseless as shown in the various studies.[2,21] The term “contact” may also signify that regarding pathogenesis this is similar to contact dermatitis, which again is not true. The term “occupational leukoderma” may be misleading as the majority of cases in the large study[2] were induced by nonoccupational household objects. Thus, “chemical leukoderma” remains as more rational and justified term to describe the disease.

## Chemical Vitiligo

Some patients who, despite the omission of all contributory toxic chemicals for more than 1 year, still develop vitiliginous patches in different parts of their body. These cases can be termed as “chemical vitiligo” to represent the vitiliginous process, which was switched on initially by the chemicals and continued even after stopping use of the chemicals.[2]

## Chemical Leukoderma Syndrome

A syndromic classification of chemical leukoderma similar to allergic contact dermatitis syndrome[22] has been described,[2] which can explain all the clinical features and patho-mechanisms adequately, which is termed as “chemical leukoderma syndrome (CLS).” The detail of this proposal is outlined in Table 4.

**Table 4:** Chemical leukoderma syndrome[2]

Stage I:	Chemical leukoderma only at the site of contact
Stage II:	Local spread of chemical leukoderma through the lymphatics beyond the site of contact
Stage IIIA:	Distant spread of chemical leukoderma through hematogenous spread beyond the site of contact
Stage IIIB:	Distant spread of chemical leukoderma through hematogenous spread beyond the site of contact along with systemic organ involvement
Stage IIIC?^a^:	A Systemic introduction (injection, inhalation or ingestion) other than skin contact causing chemical leukoderma with or without systemic organ involvement
Stage IV:	Distant spread of vitiligo-like patches even after 1 year of strictly withholding exposure to offending chemicals (“chemical vitiligo”)

a This stage is a hypothesis or probability; not yet proved

## Chemical Leukoderma: Clinical Diagnostic Criteria

Chemical leukoderma has to be excluded with certainty from every case of idiopathic vitiligo.[21] However, the differentiating factors between chemical leukoderma and vitiligo often remain clinically obscure, which often leads to misdiagnosis of chemical leukoderma syndrome (CLS) as vitiligo. The clinical criteria of diagnosis of chemical leukoderma have not yet been specifically outlined. The proposed clinical diagnostic criteria of chemical leukoderma has been outlined in Table 5.

**Table 5:** Clinical diagnostic criteria of ‘chemical leucoderma syndrome’[2]

Acquired vitiligo-like depigmented lesion(s)
History of repeated exposure to specific chemical compounds
Patterned vitiligo-like macules conforming to site of exposure
Confetti macules

Any three of the above four criteria should be present to diagnose a case of chemical leucoderma

## Conclusion

As in developing countries like India the patent loads of chemical leukoderma have been increasing in recent years and this skin ailment resembles vitiligo very closely causing psycho-social reaction, the clinico-etiological diagnosis of this skin disorder is very much warranted. Further, clinico-etiological studies can refine the diagnostic pattern as well as pinpoint etiological factors.
